# Interviewee Transcript Review: assessing the impact on qualitative research

**DOI:** 10.1186/1471-2288-9-47

**Published:** 2009-07-06

**Authors:** Victoria Hagens, Mark J Dobrow, Roger Chafe

**Affiliations:** 1 Cancer Services and Policy Research Unit, Cancer Care Ontario, Canada; 2 Department of Health Policy, Management and Evaluation, University of Toronto, Canada

## Abstract

**Background:**

This paper assesses interviewee transcript review (ITR) as a technique for improving the rigour of interview-based, qualitative research. ITR is a process whereby interviewees are provided with verbatim transcripts of their interviews for the purposes of verifying accuracy, correcting errors or inaccuracies and providing clarifications. ITR, in various forms, is widely used among qualitative researchers, however there is limited methodological guidance on how it should be employed and little is known about its actual impact on the transcript, the data, the interviewee or the researcher.

**Methods:**

ITR was incorporated into a qualitative research study in which 51 key informant interviews were conducted with a range of senior stakeholders within the Canadian health care system. The changes made by interviewees to their transcripts were systematically tracked and categorized using a set of mutually exclusive categories.

**Results:**

The study found that ITR added little to the accuracy of the transcript and may create complications if the goal of the researcher is to produce a transcript which reflects precisely what was said at the time of the interview. The advantages of ITR are that it allows interviewees the opportunity to edit or clarify information provided in the original interview, with many interviewees providing corrections, clarifications, and in some cases, adding new material to their transcripts. There are also potential disadvantages, such as a bias created by inconsistent data sources or the loss of data when an interviewee chooses to remove valuable material. The impact of ITR on the interviewee may be both positive and negative, depending on the specific circumstances and the nature of the study. The impact of ITR on the researcher was minimal in this study, but is again subject to specific circumstances of the research context.

**Conclusion:**

While ITR is employed by many researchers across numerous fields, the advantages of its use may be relatively small in terms of verifying the accuracy of qualitative interview transcripts. Researchers are advised to carefully consider both the potential advantages and disadvantages of ITR outlined in this paper before deciding to incorporate the practice within their qualitative study designs.

## Background

As part of the qualitative research process, researchers in a variety of fields, such as health [1-3], education[4,5], management[6,7] and social theory[8,9], often invite interviewees to review transcripts of their interviews. We refer to this practice, in its various forms, as interviewee transcript review (ITR). ITR can include requests for interviewees to identify and correct transcription errors or omissions, and in some cases, to clarify or provide additional information and insights directly linked to interview responses. In contrast to approaches for validating research findings, such as member checking [10-12] or triangulation [12,13], we focus on ITR as a methodological approach for verifying the accuracy of the interview transcript before it is formally coded and analyzed.

While there is a large body of literature examining ways to improve methodological rigour in the collection and analysis of qualitative data[11,14-17], there is surprisingly limited guidance regarding the verification and review of interview transcripts before they are subjected to formal coding and analysis. ITR raises a number of interesting methodological issues. Perhaps most importantly, although ITR is regularly used, it is unclear what its actual impact is on qualitative research. The purpose of this paper is to provide researchers with guidance about whether ITR should be incorporated into their study design. We conducted a detailed audit of the impact of ITR that was incorporated into the methodology for a recent interview-based, qualitative research study. Our examination focuses on four key questions regarding the impact of ITR on (1) the quality of the interview transcript (e.g., does ITR result in a transcript that more accurately reflects the verbal interview exchange); (2) the quality of the interview data derived from the interviewee (e.g., does ITR result in additional insights or clarification on what the interviewee knows or believes beyond what was exchanged during the interview); (3) the interviewee; and (4) the researcher?

## Methods

As part of an embedded multiple case study that examined the development of population-based colorectal cancer screening policies in five Canadian provinces, we incorporated ITR into the study design to assess its impact on the four questions outlined above. The study included 51 semi-structured interviews conducted with government representatives, health system leaders, clinical and epidemiological experts, and advocacy group representatives. Each interviewee signed an informed consent form before participating in the interview. A standard interview guide was used for the interviews, which were all conducted by one of the authors (VH). The interviews lasted on average 50 minutes. Each interview was digitally recorded and a verbatim transcript was prepared by a professional transcriptionist.

Interviewees were informed at the conclusion of the interview that the interview transcript would be sent to them so that it could be reviewed and that they could make corrections if necessary or desired. Each interview transcript was compared to the digital interview recording by one of the authors (VH), before being emailed to the interviewee with an invitation to "review it and send any corrections." Interviewees were asked to return any comments or feedback on their transcripts within two weeks. All edits, additions and omissions made to the interview transcript were documented. Table 1 outlines the ITR process employed.

**Table 1:** Interviewee Transcript Review process and audit

Step	Description
**1**	Signed informed consent provided; interview conducted and audio recorded

**2**	Verbatim transcript of recorded interview prepared by professional transcriptionist (transcript version 1)

**3**	Verbatim transcript reviewed by interviewer/researcher with corrections made where necessary (transcript version 2)

**4**	Verbatim transcript sent by email to interviewee along with invitation to review

**5**	Verbatim transcript reviewed by interviewee (transcript version 3)

**6**	Verbatim transcript returned by email to the interviewer

**7**	All interviewee edits/additions to the interview transcript documented and categorized by interviewer/researcher

Six mutually exclusive categories were developed which encompass all types of interviewee edits/additions/omissions made to the interview transcript (Table 2 provides examples). The categories are:

**Table 2:** Examples of transcript edits, additions and omissions by category

	Transcript sent to interviewee	Revised transcript returned to researcher
**Category 1**. Specific transcription errors/omissions corrected	**Interviewee**: That's when Dr [name not clear – recording muffled] informed the expert group that the analysis was misguided.	**Interviewee**: That's when Dr ***Smith ***informed the expert group that the analysis was misguided.

**Category 2**. Specific details added to transcript	**Interviewee**: That's when Dr, ummm, I'm sorry, I can't recall her name at the moment, but that is when she informed the expert group that the analysis was misguided.	**Interviewee**: That's when Dr ***Smith ***informed the expert group that the analysis was misguided.

**Category 3**. Specific transcription details corrected/changed	**Interviewee**: That's when Dr Smith informed the expert group that the analysis was misguided.	**Interviewee**: That's when Dr ***Jones ***informed the expert group that the analysis was misguided.

**Category 4**. Grammatical changes or minor clarifications made to transcript	**Interviewee**: Ahhhhhhhhh, ummm, ummm, that's, umm, when Dr Smith told us that our, umm, analysis was misguided.	**Interviewee**: ***That's when Dr Smith informed the expert group that the analysis was misguided***.

**Category 5**. Statements removed from transcript	**Interviewee**: That's when Dr Smith informed the expert group that the analysis was misguided.	***Section completely removed from transcript***

**Category 6**. Statements added to transcript	**Interviewee**: That's when Dr Smith informed the expert group that the analysis was misguided.	**Interviewee**: That's when Dr Smith informed the expert group that the analysis was misguided. ***I really believe that Dr Smith played an instrumental role in how we looked at the evidence supporting this policy issue***.

Category 1. Specific transcription errors/omissions corrected

These include instances where the interviewee corrected a word, phrase or name of a person, organization or other entity, data or statistic, which had either been transcribed incorrectly or left incomplete because neither the transcriptionist nor the researcher in conducting initial transcript verification were able to accurately interpret what was said.

Category 2. Specific details added to transcript

These include instances where the interviewee added a specific detail to the transcript, such as the name of a person, organization or other entity, date or statistic, that the interviewee had not been able to recall at the time of the interview.

Category 3. Specific transcription details corrected/changed

These include instances where a specific detail that had been provided in the interview, such as the name of a person, organization or other entity, date or statistic, was corrected or changed by the interviewee upon review of the interview transcript.

Category 4. Grammatical changes or minor clarifications made to transcript

These include instances where the interviewee rephrased a statement made in the interview. The meaning of the statement was not changed, but edits were made to grammar or sentence structure, or the meaning was conveyed using different vocabulary.

Category 5. Statements removed from transcript

These include instances where an interviewee removed a specific section of the transcript.

Category 6. Statements added to transcript

These include instances were an interviewee added comments or statements to the transcript to offer new perspectives and insights which were not made in the interview.

One author (VH) reviewed all revised interview transcripts and classified all edits/additions/omissions into one of the six categories.

## Results

Of the 51 interviewees invited to review their interview transcripts, 22 (43%) responded either by returning a revised transcript (16) or by indicating that they had reviewed the transcript but had no revisions to make (6). Of the 16 interviewees who submitted revisions to their transcripts, 12 did so by sending a revised transcript to the researcher by email with changes tracked using the word processor's "track changes" feature. Two interviewees submitted printed copies of the transcript with hand-written revisions, while another two interviewees submitted electronic copies of the transcript with changes made but not tracked. Table 3 summarizes the results of the ITR audit for the 22 interviewees who indicated that they had reviewed their interview transcripts.

**Table 3:** Interviewee Transcript Review results by edit/addition/omission category

		Category 1	Category 2	Category 3	Category 4	Category 5	Category 6
		
Interview #	Revisions made	Specific transcription errors/omissions corrected	Specific details added to transcript	Specific transcription details corrected/changed	Grammatical changes/minor clarifications made to transcript	Statements removed from transcript	Statements added to transcript
04	Yes	1	-	-	-	-	-

05	No	-	-	-	-	-	-

07	Yes	8	2	-	12	-	2

08	Yes	-	1	-	21	16	3

09	Yes	-	-	-	4	2	3

10	Yes	-	1	-	-	-	-

14	Yes	3	1	-	2	-	-

15	Yes	1	1	2	2	2	-

16	Yes	-	1	-	51	7	13

21	Yes	3	-	2	1	-	-

29	Yes	4	-	1	15	1	13

31	Yes	4	-	-	-	-	-

32	Yes	1	1	-	45	-	5

33	Yes	7	1	-	5	-	-

36	No	-	-	-	-	-	-

37	Yes	1	2	-	12	-	4

38	Yes	6	-	1	34	10	-

40	No	-	-	-	-	-	-

41	No	-	-	-	-	-	-

49	Yes	-	-	-	-	1	-

50	No	-	-	-	-	-	-

51	No	-	-	-	-	-	-

**TOTAL**	16/6	39	11	6	204	39	43

For the 16 revised transcripts, 39 specific transcription errors were corrected (category 1), 11 specific details were added (category 2), and 6 specific transcription details were corrected (category 3). There were 204 instances in which an interviewee made grammatical changes and/or revised the transcript in an apparent effort to clarify or articulate better a point made in the interview (category 4). There were another 39 instances where interviewees removed statements from their transcripts (category 5). These were generally statements of opinion, although a few factual statements were also removed. There were also 43 instances where interviewees added new information that had not been discussed during the interview (category 6). Two of the interviewees added numerous new and substantive comments that had not been discussed during the interview.

## Discussion

The audit provided useful data for assessing the potential advantages and disadvantages of ITR. Table 4 summarizes the impacts of ITR on the transcript, the data provided, the interviewee and the researcher.

**Table 4:** Impact of Interviewee Transcript Review

	Advantages	Disadvantages
**Impact of ITR on the transcript**	▪ Additional transcription errors and omissions not identified and/or corrected by the researcher can be corrected by interviewees through ITR.	▪ Interviewees may revise the transcripts in such a way that they no longer accurately reflect the verbal exchange during the interview.

**Impact of ITR on the data**	▪ Missing details can be added. ▪ Incorrect details can be corrected. ▪ Articulation of important points can be improved. ▪ Opportunity for interviewee to provide additional insights.	▪ Candid responses to interview questions may be more valuable than responses which have been edited. ▪ Interviewees may remove valuable material from the transcript.

**Impact of ITR on the interviewee**	▪ Enables the rights of the interviewee.	▪ Time and effort required to participate in ITR ▪ Emailing transcripts increases the risk of third party viewing. ▪ Reviewing verbatim transcripts can make interviewees feel uncomfortable (e.g. grammatical concerns).

**Impact of ITR on the researcher**	▪ Reinforces the relationship between the researcher and interviewee.	▪ Increased time and effort for data preparation and communication with interviewees.

### Impact of ITR on the interview transcript

While there is some disagreement among researchers about what constitutes good quality transcription [16,18], for the purposes of this audit we characterize a high quality interview transcript as a complete and accurate reflection of the verbal exchange between the interviewer and interviewee during the interview. Based on this characterization, transcription quality was improved by ITR (beyond normal transcript verification by the researcher) with 39 errors and omissions corrected (category 1). Typically, however, these were relatively minor corrections and while all interviewees were offered the opportunity to revise their transcripts, only 16 (31%) interviewees returned a revised transcript, with some of these interviewees stating that they did not read their transcripts carefully because they found them to be too lengthy and/or cumbersome to review. As well, the majority of interviewees who revised their transcripts did so in such a way that the transcript no longer reflected accurately the verbal exchange during the interview. Transcripts revised by interviewees therefore can be seen to represent a different type of data source than the transcripts not revised by other interviewees.

The incremental benefits of ITR in producing marginally higher quality transcripts likely do not justify its use. If the goal is solely to correct inaccuracies in interview transcripts then a modified process involving researcher verification of the interview transcript, as was also done in this study, followed by targeted interactions with interviewees to clarify any outstanding errors, inaccuracies or omissions, is likely a more appropriate approach than ITR. In our study, having one of the researchers check the transcript provided by the transcriptionist against the audio recording corrected the vast majority of transcription errors and omissions. A researcher could then contact specific interviewees with specific questions about their transcripts (such as the correct spelling of a name, or a key word that is missing from a sentence), rather than sending full transcripts to all interviewees for their review. Combined, researcher review and targeted follow-up can improve transcription quality without requiring interviewees to review their full transcripts.

### Impact of ITR on the interview data

The impact of ITR on the quality of interview data is a more complicated issue. An accurate interview transcript can still represent poor quality data if the interviewee has inaccurately conveyed his or her knowledge or beliefs. Existing guidelines and methods protocols which address quality and rigour in qualitative research provide only limited and indirect guidance on how ITR techniques impact on data quality[10,19-22]. Based on the results of this audit, ITR can improve the quality of interview data where specific details are added or corrected by interviewees such as dates, statistics, and the names of people, organizations or other entities (categories 1, 2, and 3). Almost two-thirds of the interviewees who reviewed their transcripts within our audit added or corrected at least one such detail. While specific missing details may be more reliably added through direct, targeted communication with interviewees, incorrect details are most likely only detectable by the interviewees themselves and so ITR may be the only way to achieve this improvement to data quality.

It is less clear whether interviewees should be given the opportunity to edit the wording of statements made in their interviews (category 4) or to include additional information (category 6). Such results can improve data quality by providing researchers with increased clarity around key statements in the interview and/or providing additional insights which would not otherwise have been acquired. Indeed, in this study, the ITR process resulted in substantive new insights from two interviewees which could potentially influence the data analysis. However, there are important questions which need to be considered regarding whether ITR is the appropriate approach to obtaining these additional insights. First, it may be argued that spontaneous responses delivered in an interview setting are likely to offer different insights than responses modified through ITR. Even where the intention of an interviewee is simply to clarify a statement by editing grammar or sentence structure, information which was gleaned from a more candid expression during an interview might be lost[23]. Second, incorporating interviewees' key additional comments and revisions may generate a systematic bias, with some interview transcripts reflecting interviewees' more thoughtful and time-considered responses to interview questions, compared with other transcripts simply reflecting the unaltered verbal interview exchange. And third, it should be noted that employing ITR in our study resulted in only two of the 51 interviewees (two of 22 interviewees who responded to the ITR request) providing significant new insights, creating questions regarding the efficiency of this approach for collecting additional data.

ITR also impacts on interview data quality when data are retracted by interviewees[24]. In this study, seven interviewees removed at least one statement from their transcripts, with two of those interviewees removing statements which provided particularly important and/or controversial comments. This study involved discussions regarding sometimes sensitive issues surrounding the development of a major public policy decision and some candid interview comments were particularly informative. However, based on the ethics protocol for the project, statements removed by interviewees were not included in the analysis. While data loss can be viewed as a potential disadvantage of ITR, the ethical responsibility to protect the interviewee can be enhanced through the process.

### Impact of ITR on the interviewee

For interviewees, an advantage of ITR is that their rights as research participants are reinforced. The right to withdraw one's own responses from a research study is a right which is ensured to research participants in many ethics protocols and was stated in the consent forms signed by interviewees in this study. However this right is often difficult to exercise if the participant is not provided with a transcript or summary of the interview exchange. As shown above, seven of our interviewees chose to withdraw material from their transcripts, thus exercising a right that they may not otherwise have had the opportunity to employ.

This advantage for the interviewee needs to be balanced against some potential disadvantages. Serious harm could be caused if a transcript is mistakenly sent to the wrong interviewee. While necessary care was taken to ensure that this did not occur in this study, it is important to note that a breach of privacy of this sort could result in harm to participants greater than any benefits gained from ITR, as well as a violation of the protections researchers make to their study participants. Additionally, some interviewees may feel discomfort reviewing verbatim transcripts. In particular, when the interview focuses on personal experiences and/or sensitive topics, requests for the interviewee to review the transcript may add considerably to the burden of participation. Interviewees may even experience discomfort with respect to reviewing poor grammar reflected in their transcripts, as was expressed by several interviewees in our study.

Finally, it is important to note that considerable time and effort is required from interviewees to participate in ITR. Transcripts can be both lengthy and cumbersome to read, and although participation in transcript review is voluntary, interviewees who wish to participate or who perceive pressure to participate may be negatively impacted by the time and effort required.

### Impact of ITR on the researcher

The impacts of ITR on the researcher should also be considered in the context of weighing the overall advantages and disadvantages of the technique. One impact of ITR on the researcher is that the relationship between interviewees and researchers is reinforced. Lines of communication are kept open, and in some cases an additional method of correspondence is established (e.g., email), thus facilitating future exchanges. In this study, several interviewees indicated during their interview that they had relevant documents that might be of interest to the researchers, with the ITR process providing a convenient follow-up mechanism. Additionally, ITR can save the researcher time required to fill-in blanks and/or check questionable details within transcripts.

One potentially negative impact of ITR on the researcher is the additional time and effort required for transcript preparation and communication with interviewees. Appropriate steps must be taken to securely deliver the transcript to the appropriate interviewee and analysis of interview data can be delayed due to the time required to allow interviewees to review their transcripts. For this study, the two-week time window for interviewees to respond with transcript revisions did not alter our analysis timelines significantly, however other research processes may be subject to different time pressures thus altering the impact of this delay.

### Study limitations

The impacts of ITR likely vary by the target population and the nature of the research questions addressed. In this study, we conducted interviews with key stakeholders in the Canadian health care system, including senior clinical and administrative leaders. Issues related to power differentials between the researcher and the interviewee, literacy levels, or email access were not a factor for our interviewee population. Therefore, while the process of ITR was well within the basic capabilities of our study population, it might not necessarily be so for other more varied populations, particularly patient populations. The nature of our project directed focus on interviewees' perceptions of key events and factors within a complex policy-making process. Other issues may arise when interviews are primarily intended to capture interviewees' experiential knowledge, where contradictions and factual misinformation may provide the researcher with significant insights.

It should be noted that in our assessment of the impacts of ITR we did not attempt to assess the various methods of conducting ITR. Recognizing that there are numerous variables in this process – such as when and how the interviewees are invited to participate in ITR; whether transcripts are provided to all interviewees in a study or only those interviewees who respond positively to an ITR invitation; and the specific instructions given to interviewees on the purpose of ITR and on the types of feedback they are requested or invited to provide – further research is required to assess the role played by these variables in the impacts of ITR on the transcript, the data, the interviewee and the researcher.

## Conclusion

The decision to use ITR must weigh its potential advantages and disadvantages as they relate to the particular study at hand. While ITR is employed by many researchers across numerous disciplines, overall our audit revealed that the advantages to its use may be relatively small, particularly in relation to the added time and effort required for interviewees and in light of other existing techniques to address transcript and data quality such as researcher review of transcripts and targeted communication with interviewees. Furthermore, ITR as defined in this study, is not intended for the validation of qualitative research findings. However, further examination of how ITR could be potentially integrated with established methods for validating qualitative research findings, such as member checking, is needed. Researchers who are considering the use of ITR are advised to be clear on what they hope to achieve through its use and to take into account the various impacts associated with ITR for their specific project and interviewee population.

## Competing interests

The authors declare that they have no competing interests.

## Authors' contributions

All authors participated in the conception and design of the study. The key informant interviews and ITR process were carried out by VH. The audit was conducted by VH and MJD. VH prepared the original draft of the manuscript, and all authors reviewed and critically revised the original and subsequent manuscript drafts and approved the final manuscript.

## Pre-publication history

The pre-publication history for this paper can be accessed here:

http://www.biomedcentral.com/1471-2288/9/47/prepub
